# Dry Matter Losses and Methane Emissions During Wood Chip Storage: the Impact on Full Life Cycle Greenhouse Gas Savings of Short Rotation Coppice Willow for Heat

**DOI:** 10.1007/s12155-016-9728-0

**Published:** 2016-04-13

**Authors:** Carly Whittaker, William Macalpine, Nicola E. Yates, Ian Shield

**Affiliations:** Agro-ecology Department, Rothamsted Research, Harpenden, Hertfordshire AL5 2JQ UK

**Keywords:** Life cycle assessment, Storage, Short rotation coppice willow, Losses

## Abstract

A life cycle assessment (LCA) approach was used to examine the greenhouse gas (GHG) emissions and energy balance of short rotation coppice (SRC) willow for heat production. The modelled supply chain includes cutting multiplication, site establishment, maintenance, harvesting, storage, transport and combustion. The relative impacts of dry matter losses and methane emissions from chip storage were examined from a LCA perspective, comparing the GHG emissions from the SRC supply chain with those of natural gas for heat generation. The results show that SRC generally provides very high GHG emission savings of over 90 %. The LCA model estimates that a 1, 10 and 20 % loss of dry matter during storage causes a 1, 6 and 11 % increase in GHG emissions per MWh. The GHG emission results are extremely sensitive to emissions of methane from the wood chip stack: If 1 % of the carbon within the stack undergoes anaerobic decomposition to methane, then the GHG emissions per MWh are tripled. There are some uncertainties in the LCA results, regarding the true formation of methane in wood chip stacks, non-CO_2_ emissions from combustion, N_2_O emissions from leaf fall and the extent of carbon sequestered under the crop, and these all contribute a large proportion of the life cycle GHG emissions from cultivation of the crop.

## Introduction

With the implementation of the Renewable Energy Directive (RED), there has been significant growth in the uptake of renewable energy in Europe [1]. Renewable sources currently provide 14.1 % of the European (EU_28_) energy supply [2], although the overarching target is to generate 20 % by 2020 [3]. Biomass could contribute up to two thirds of the target [4]: equivalent to approximately 124 million tonne of oil equivalent (Mtoe) [5]. By 2020, a total of 19.3 million ha of agricultural land could be diverted to dedicated bioenergy production to provide 100 Mtoe of energy, while complying with good agricultural practice and without significantly affecting domestic food production [6, 7]. Additionally, 40 Mtoe of forest biomass are envisaged to be available to biomass energy systems by 2020 without compromising environmental criteria [6]. Over the last decade, a strong forestry sector and competitive pricing has meant that Europe has been the prime market for energy-related biomass trade, particularly for wood chips and pellets [8].

Wood chip supply chains involving forestry or coppice consist of cultivation, harvesting, chipping, storage and transportation, though often, the material can be harvested in chip form. Freshly harvested biomass often has a moisture content (MC) of 50 % [9], and although it is possible to utilise fuel up to 65 % MC in modified furnaces, it is beneficial to dry the material to increase the net calorific value of the biomass [10]. This often occurs outdoors in piles, which allows it to dry by redistributing the moisture within the biomass, resulting in a wet outer surface and a drier inner part [11]. The outer parts of the stack can dry due to evaporation, and in theory, the biomass can reach 25 % MC by the end of summer [9].

One of the most difficult tasks in biomass harvesting is how to manage the storage of the material to reduce material losses due to degradation (Wihersaari 2005). Wood is a biologically active material, and unlike fossil fuel, it undergoes changes during storage [10]. It is typical for the temperature in wood chip stacks to rise very rapidly as the material starts to decay, and in extreme cases, spontaneous combustion can occur [12]. Such a temperature change is a sign of microbial decomposition [9, 13], which can lead to material and energy losses [12]. A review of literature shows that dry matter losses can range between 1 and 27 % for the whole storage process (Table 1). More recent studies are showing some results consistent with the higher rate of loss: A whole heap dry matter loss of 21 % was observed in willow chip after 3 months of storage in the UK [27], and over 9 months, a DM loss of 22 and 21 % was found in covered heaps of fine and coarse poplar chips, respectively [28]. The impact that these dry matter losses have on the greenhouse gas (GHG) emission savings and net energy yield on wood chip systems has not been explored in detail in the literature. Some studies have explored the impact of dry matter losses in bioethanol supply chains but focus on baled feedstocks and silage [29, 30]. The studies showed that dry matter losses can increase the GHG emissions of cellulosic bioethanol by up to 53 %, depending on the storage method used. The aim of this study is to examine the impact of losses in the context of the full supply chain, using short rotation coppice (SRC) willow as an example. This will be examined following a life cycle assessment (LCA) approach, using data that is representative of current commercial systems. The LCA model will be used to quantify the relative impacts of dry matter losses and changes in moisture content on the GHG emissions from SRC supply chains.

**Table 1:** Literature review of dry matter losses and moisture content changes during outside storage of wood chips

		Storage conditions	Moisture content (% w.b)		Ash content (% dm)		Lower heating value (MJ/kg)		Dry matter loss during storage	
Reference	Wood type		Before storage	After storage	Before storage	After storage	Before storage	After storage	% During storage	% Per month
(Wihersaari [14])	Generic wood	Stored in heaps	–	–	–	–	–	–	1–2	–
(Afzal et al. [15])	Forest residue chips	3 m high cone-shaped heaps, from July 2007 to August 2008:								
		Covered with tarp	59.5	20	0.43	1.06	19.6	19.56	8	0.6
		Built on forest floor	59.5	160	0.43	1.09	19.6	19.44	27	2.1
		On plastic sheath	59.5	160	0.43	1.12	19.6	19.28	22	1.7
(Jirjis [16])	Poplar chips	Stored in bins (3 × 2.3 m) with:								
		Drying with continuous ventilation	57	19.3					3.5	1.3
		Cooled to 1–4 °C	57	42					7.1	2.7
		Cooled to 20–35 °C	57	47.7					7.9	3.0
		No cooling	57	44.9					19.1	7.2
(Pari et al. [17])	Poplar chips	Stored in piles for 8 months	62.9	50					10	1.3
(Eriksson [18])	Birch chips	1 month, summer storage	32.8	30.2					3.6	3.6
		1 month, summer-winter storage	48.8	40.8					11.3	1.6
(Lamond et al. [19]) in (Garstang et al. [20])	Sitka spruce	Stored in a sealed bin								1
		Stored in an unsealed bin								2
(Buggeln [21]) in (Garstang et al. [20])		Outside chip storage, ‘rule of thumb’								1
(Gjølsjø [22]) in (Garstang et al. [20])	Birch chips	Small piles							9.7	1.2
		Large piles							7.5	0.9
(Manzone et al. [23])	Poplar chips	Uncovered piles	45	30					9.8	1.6
		Piles covered with black plastic	45	55					5.1	0.9
		Piles covered with fleece	55	21					9.3	1.6
		Piles covered with roof	40	22					7.1	1.2
(Barontini et al. [24])	Poplar chips	Stemwood chips, piled outside for 6 months	47.7	33.6	3.02	3.2	14.34	16.35	26	4.3
		Crown wood chips, piled outside for 6 months	54.2	30.3	2.91	3.09	15.02	15.87	10	1.7
(Jirjis et al. [25])	Poplar chip	Ventilated piles	50.6	61.7	0.51	4.1	15.4	17.73		
		Uncovered piles	64.4	39.2	0.51	11.7	15.4	16.13		
		Covered piles	60.3	59.14	0.51	2.9	15.4	18.29		
		Compacted pile	62.9	50	0.51	3.4	15.4	18.03		
(Thörnqvist [9])	Forest residue chips	Stored in piles between April and August	50	20				3 % loss	11	0.3
(Jirjis [26])	Willow chips	Stored in 3 m piles	52–55 %	53 %	2	2	19.57	19.48		
		Stored in 6 m piles	52–55 %	43 %	2	1.89	19.57	19.44		

Another issue of concern that is addressed here are the GHG emissions that arise from the storage of wood chips. It is possible that wood stacks undergo composting, as it contains some readily available carbohydrates that can be fermented to lactic acid, volatile fatty acids and alcohols, with the release of carbon dioxide and heat [31]. The cellulose and hemicellulose components of wood can be degraded by a broad spectrum of fungi and bacteria [11]; however, at least 18 % of it is recalcitrant to degradation because of its close association with lignin [32]. It is suggested that the decay process can lead to the release of methane [14]. In the Intergovernmental Panel on Climate Change (IPCC) Guidelines, emissions of methane from windrow composting are reported to be between 0.08 and 20 g CH_4_/kg waste composted, assuming the waste has a moisture content of 60 % and 25–50 % degradable carbon [33]: corresponding to a conversion of 0.01–6 % of the carbon in the biomass to methane. A review of literature and windrow compost trials showed methane emissions of between 0.04 and 2.2 g/kg fresh material [34], which represents less than 1 % of the carbon present in the compost material. Similar records were made by Sommer and Møller [35]. Beck-Friis et al. [36] estimated that 4–5 % of the carbon left compost heaps in the form of methane.

Wihersaari [14] theorised that wood chip storage could result in emissions of methane, and applying standard emission factors to a storage of forest biomass has been shown to compromise the GHG emission achieved from utilising such biomass [37]. Experimental studies, however, show variable results. A recent study showed that methane concentrations in a willow heap peaked at around 400 ppm after 50–60 days, but it was not known whether this resulted in a fugitive emission from the stack [27]. Samples from gas probes embedded in a pine woodchip stack in Ferrero et al. [38] showed that carbon dioxide was the only GHG present in appreciable concentration. Pier and Kelly [39] detected methane concentrations of 4–63 % across probe samples in a sawdust pile, with methane contributing 20 % of the gas emitted from the stack. He et al. [40] studied small (2.5 kg) samples of forest residues and detected methane concentrations of 0.15 % in the headspace, which was small, but constant for up to 25 days. Their results suggest that the conditions within the wood pile are not favourable for methanogenic bacteria; however, it is possible that a slow emission of methane occurs from wood storage piles.

It is also hypothesised that there are emissions of nitrous oxide from wood chip stacks. Such emissions can result from the activity of nitrifying or denitrifying bacteria that utilise nitrogen derived from bark, cambium and foliage [10]. Estimates in literature indicate that the emissions of N_2_O–N can be between 0.5 and 0.7 % of the total initial nitrogen present in the biomass [14]; however, it is believed that the temperature increases observed within the stacks are inhibitive to the bacteria involved [36]. As a result, emissions of nitrous oxide are not as great a concern as methane emissions [14].

This study will use the results of the LCA of SRC to investigate the potential impacts of GHG emissions from outside storage on the GHG emission savings achieved by the system. To provide a reference case in order to quantify GHG emission savings, a case study involving the use of SRC willow for heat generation will be examined and compared with GHG emissions from conventional heating fuels.

## Methods

The GHG emissions from the cultivation, harvesting and utilisation of short rotation coppice were examined following a life cycle assessment approach. This is performed using an MS Excel-based model, and the following sections describe it. The LCA is performed according to the principles described in the ISO 14040 [41].

### Goal and Scope

The goal of the study is to evaluate the GHG emissions that arise from the use of SRC willow chips for heating and to test the sensitivity of the overall GHG emission savings to methane emissions and dry matter losses during the wood chip storage phase. The functional unit is 1 GWh delivered heat from SRC chips. The final unit of measurement will be kg CO_2_ eq./GWh heat.

**Fig. 1:**
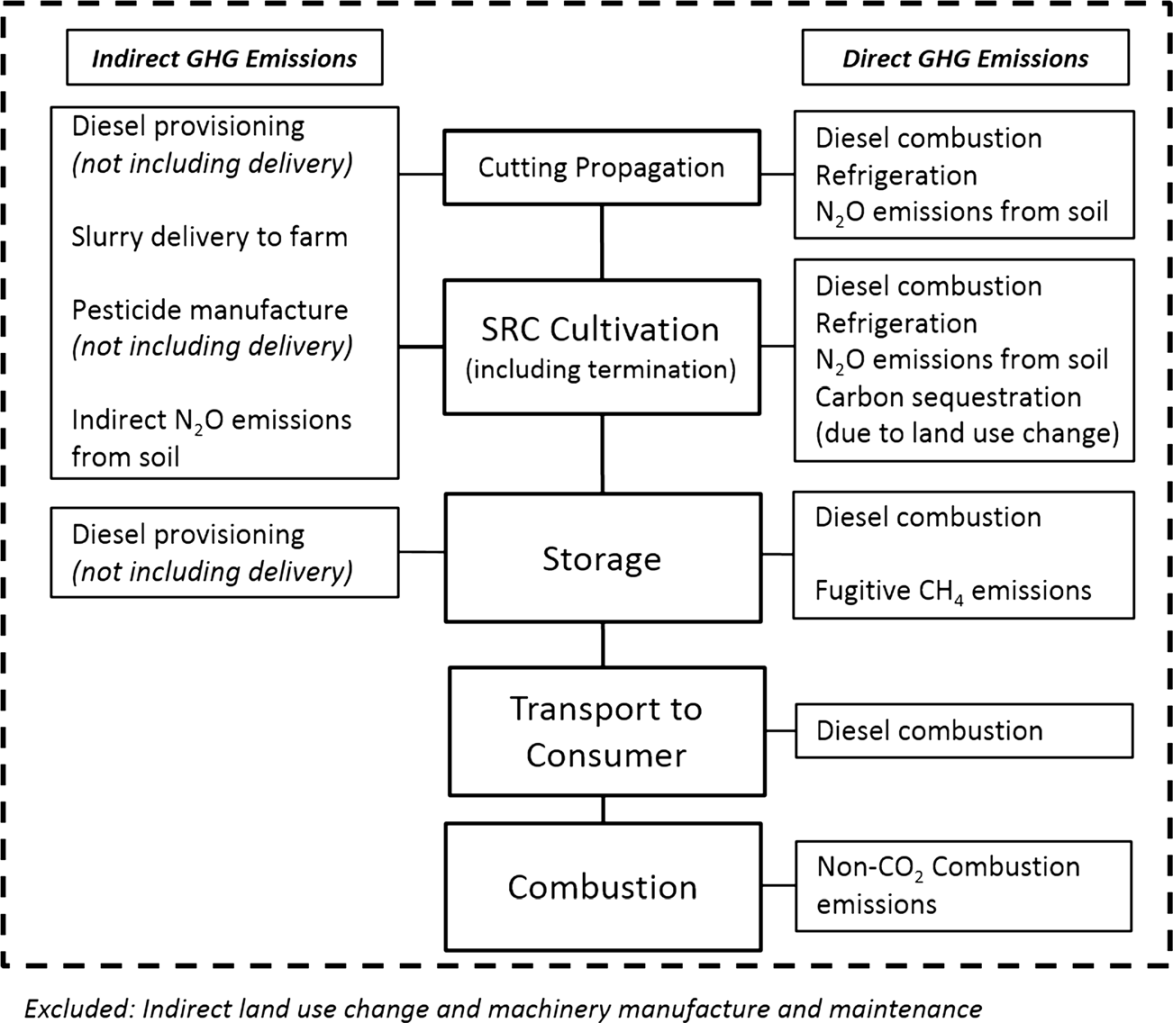
System boundaries of the LCA study, including direct and indirect sources of GHG emissions

The scope of the study includes cutting production, main crop site establishment, agronomy, harvesting, delivery to storage, outside storage, transportation and combustion (Fig. 1). The system boundaries of the study include cuttings, diesel fuel consumption, fertiliser application and pesticide use. As the main fertilisation is provided from manure, the delivery of this to the farm is included. Also, refrigerated transportation of rhizomes to the site is included. Delivery to the farm of other materials (diesel, pesticides) is not included, though emissions from provisioning diesel (through refining, etc.) and pesticide manufacturing are included. The study includes carbon sequestration under the crop due to direct land use change from agricultural land, though due to uncertainties, this is examined separately. Indirect land use change is not examined. Machinery manufacture is not included as this is expected to be small contribution (<4 %) [42], and fencing is excluded as it is prohibitively expensive [43]. Such inputs are usually excluded from GHG emission reporting (e.g. the RED [3]).

### Inventory Data

The inventory data used in the LCA study is described here according to the different life cycle stages of the willow crop. The data is based on the best available knowledge of production in the UK. The cropping system is representative of typical current commercial SRC used for heat or power purposes. Approximately 3000 ha of SRC are currently grown for bioenergy in England [44]. Some LCA studies examining SRC do not provide transparent accounts of the diesel fuel consumed during cultivation [45, 46], and some [47, 48] use theoretical equations involving machine power, power take off and work rate to estimate fuel consumption rates of machinery. This method has been shown to overestimate fuel consumption rates so could be used as a conservative estimate if other data is lacking [49]. Here, the consumption of diesel is collected from the literature and cross-referenced with an industry expert (Table 2).

**Table 2:** Summary of fuel consumption data for SRC cultivation, harvesting and termination over the lifetime of the crop

Year	Phase	Operation	Fuel consumption range (l/ha)			
			Low	Reference	High	Reference
0	Establishment	Ploughing	17	Mouldboard ploughing (Lal [50])	28 [124]	Mouldboard ploughing (Lal [50])
		Power harrow	4	Nemecek et al. [51]	20	Heavy work harrowing (Lewandowski et al. [23])
		Planting	13	Matthews et al. [52]	15	Newer planters are more powerful (industry expert)
		Rolling	2	Williams et al. [53]	3	Nemecek et al. [51]
		Spraying	1	Lal [50]	3	Heller et al. [47]
		Total	37		69	
1	Maintenance	Brush cutter	4	Matthews et al. [52], Heller et al. [47]	6	Newer brush cutters are more powerful (industry expert)
		Slurry application	8	Heller et al. [47]	12	Williams et al. [53]
		Spraying	–		–	
		Total	13		21	
Every 3 years	Harvesting biomass	Forage harvesting	75		75	Contractor (Styles and Jones [46])
		Tractor and trailer offsite	10		10	Estimate
		Total	85		85	
23	Termination (main crop)	Stump mulching (two passes)	360		360	Eriksson and Gustavsson [54]
		Herbicide spraying	–		–	
		Total	361		363	

#### Cutting Multiplication

Cuttings are propagated in multiplication beds, at densities of 40,000 stools/ha. Material for propagation is harvested as 1-year-old stems when plants are dormant in January/February. It is estimated that each stool yields three marketable rods, which are cut with rod harvesters. The resulting rods are trimmed with circular saws to 2-m lengths, which will later be planted as 20-cm cuttings, giving ten cuttings per rod. Cuttings have an average weight of 30 g and diameter of 1.5 cm. Assuming a 10 % failure rate of the originally planted stools, an approximate yield of just over 1 million cuttings/ha is achieved. The rods are wrapped in plastic and stored in wooden crates and refrigerated at −2 to −4 °C until planting in February/March [43, 55]. Cuttings can also be planted as late as June, so the period of refrigeration can be up to 5 months. The methodology presented in the DEFRA Accounting Guidelines is followed to calculate the GHG emissions from refrigeration [56], refrigerated transport of cuttings [57], assuming a charge capacity of 0.5 kg, and a refrigerant with a global warming potential of 1725 kg CO_2_ eq./kg (R410A) [58]. Although cuttings can be locally produced, they can also be transported throughout the UK or even overseas. For example, the majority of the 1100 ha of SRC willow established in Northern Ireland were transported there as planting rods from multiplication beds in Markington, near Harrogate, North Yorkshire, England. A default of 100 km is used here, and it is assumed that at least 30 ha of material can be delivered in one journey; however, current planting rates are about 20 ha.

#### Main Crop Establishment

The process of site establishment requires between 37 and 69 l diesel/ha, involving ploughing, power harrowing, planting, rolling and spraying (Table 2). In a main SRC crop, planting densities of 18,000/ha are required to provide a final establishment of 15,000/ha [43]. Cutting multiplication beds are maintained for 7 years, as older stools become increasingly more difficult and hence expensive to remove. It is assumed that the main SRC crop is viable for at least seven harvests or 23 years including a preparation year and cutting at the end of the first year [46]. Estimates of the total life span of the crop range between 16 and 30 years [48, 59]. It is suggested that the very minimum time that the crop would be grown for is 5 years, as termination prior to this means that growers must repay any establishment grants received [60]. Such may be an extreme case, however, as removing a crop after 5 years would incur a substantial financial loss, and the energy crop scheme is currently closed for new applications for the foreseeable future. A sensitivity analysis is performed to determine the importance of crop life on the final GHG emissions.

#### First Year Maintenance Onwards

First year maintenance involves first year cutback, fertiliser and herbicide application, requiring between 13 and 19 l diesel/ha, from which no biomass is harvested. The first harvest is made 3 years afterwards [48]. The Farm Pocketbook [61] budgets fuel requirements for SRC harvesting at £50/ha: Assuming a red diesel cost of 66.67 p/l [62], it equates to approximately 75 l/ha, similar to other estimates [46] and confirmed by the industry expert. A tractor then requires a conservative estimate of 10 l/ha to carry the crop offsite. Willow coppice yields were based on an empirical yield model which predicts an average yield of 9 oven-dried tonnes (ODT)/ha/year once the crops are established [63]. As the crop is harvested at 50 % moisture content, this means that the quantity handled is twice as much as the yield in ODT.

#### Fertilisation and N_2_O Emissions from Soil

Willows have a low nitrogen requirement, with between 150 and 400 kg N being applied to the crop over a 23-year rotation [43] and 100 kg N/ha/year to a multiplication beds. In comparison, a milling wheat crop receives 250 kg N/ha in a single year [61]. If soils are sufficient in phosphorus and potassium, no additional inputs may be required; otherwise, some maintenance could be required [43]. It is expected that the annual, rather than tri-annual cutting cycle in the nursery crop, will increase the nitrogen demands, and nitrogen application will produce a better quality cutting. A suggested rate of 100 kg N/ha is applied after the first cut and again in the following years if necessary. The nitrogen is applied in the form of ammonium nitrate, in order to monitor N use. This may not necessarily be carried out, due to the potential cost of the operation.

In the main crop, fertiliser requirements were assumed to be met by pig slurry application, received from a local source (10 km), at a rate of 12 m^3^/ha assuming a typical N, P and K nutrient content of 5 kg N/m^3^, 1 kg P/m^3^ and 2.5 kg K/m^3^, respectively [64]. It is assumed that the manure is a waste product from animal husbandry and not allocated upstream GHG emissions from that sector. The application rate corresponds to approximately 60 kg N/ha, which is recommended in growers guides [61]. The slurry is applied after preliminary cutback and then after each 3-year harvest, so when averaged out over the whole crop lifetime, it equates to 4 m^3^/ha/year or 20 kg N/ha/year. Direct and indirect N_2_O emission rates for SRC are expected to be the same as for arable crops, as demonstrated by experimental data [65]. These are calculated using default data in the IPCC Guidelines for National Inventories [66]. It must be acknowledged that there is a high degree of uncertainty associated with the IPCC defaults, though these may be reduced using more detailed modelling, such as the DNDC model [67–69]. Such an analysis requires data on soil composition, meteorological data and detailed accounts of what fertilisers are used and when (Brown et al. 2002). There is a trade-off between the increased certainty in the GHG emission result and more detailed data collection. Another solution may be presented in the near future, when it is expected that a series of UK regional maps of local N_2_O emission factors will soon be developed (Whitaker et al. 2010).

Another source of uncertainty in the SRC life cycle is the N_2_O emissions from leaf fall, though this is examined in greater detail in more recent publications [70, 71]. Data from Rothamsted Research recorded an average annual leaf senescence in willow or around 3.8 t/ha/year. Senesced willow leaf has a nitrogen concentration of 22–28 mg/g dry matter [72]; however, the rate in which the matter decomposes will affect the net influx to soil. The N component of litter is not the fastest to decline in the leaf material compared to carbon and phosphorus [73], and only one third of the nitrogen is returned to the soil within 1 year [74]. Monitoring a SRC poplar crop showed that the majority of variation in fluxes of N_2_O was caused by land use change heavy rainfall rather than leaf fall [75, 76]. Data UK-specific data is limited, so here, the LCA model uses data from a Canadian study of willow SRC, which measured an average input rate of 20 kg N/ha/year over four sites over 4 years [77].

#### Pesticide Application

Data for pesticide application is listed in Table 3 and are based on the AFBI short rotation coppice best practice guidelines [43]. Pesticide application is intensive pre and post plating in the establishment year; however, after this period, pesticide inputs are low. Before initial cultivation, the site is sprayed with glyphosate, which can be re-applied in the early spring if weeds persist after the winter. Post planting, pre-emergent residual herbicides are used to keep the crop clean during the establishment phase and should be applied within a week of planting. At this stage, ex-grassland sites receive additional pesticides to provide *Tipula paludosa* (leatherjacket) control. Amitrole (Weedazol at 20 l/ha, 4.5 kg a.i/ha) has traditionally been applied after first year cutback but is now withdrawn from sale. These initial applications are normally sufficient, and no more herbicides are applied for the remainder of the crop’s lifetime. If initial weed control was poor, a few sprays can be used to control some weeds if they are problematic (Table 3). These are normally only applied in the establishment year, and commercially, this is rare. *Chrysomelids* (willow beetles) are occasionally a problem during the life of the plantation; however, population sizes of the pest vary considerably from year to year. Growers occasionally use an insecticide to control beetle populations if they are high, but this is not recommended for both economic and ecological reasons, plus it is difficult to spray crops that are affected after the first year after cutting due to their physical size when the above \ground biomass is older than 1 year.

**Table 3:** Full breakdown of primary energy and GHG emissions from generation of 1 MWh from SRC coppice

Stage	Process/input	Energy	Carbon dioxide	Methane	Nitrous oxide	Other GHG	GHG
		(MJ/MWh)	(kg CO_2_/MWh)	(kg CH_4_/MWh)	(kg N_2_O/MWh)	(kg CO_2_ eq./MWh)	(kg CO_2_ eq./MWh)
Establishment	Ploughing	1.41	0.09	0.00	0.00	0.00	0.09
	Power harrowing	1.00	0.06	0.00	0.00	0.00	0.07
	Rolling	0.15	0.01	0.00	0.00	0.00	0.01
	Herbicide application	0.15	0.01	0.00	0.00	0.00	0.01
	Planting	0.65	0.04	0.00	0.00	0.00	0.04
	Cutting propagation	0.06	0.01	0.00	0.00	0.00	0.02
	Cuttings (transport)	0.13	0.01	0.00	0.00	0.09	0.21
	Cuttings (refrigeration)	0.00	0.00	0.00	0.00	0.11	0.11
	Agrochemicals	0.78	0.01	0.00	0.00	0.00	0.02
Total establishment		4.33	0.25	0.00	0.00	0.20	0.58
First year cutback and fertilisation	Herbicide application	0.15	0.01	0.00	0.00	0.00	0.01
	Slurry spreading	0.40	0.03	0.00	0.00	0.00	0.03
	Mowing	0.20	0.01	0.00	0.00	0.00	0.01
	Agrochemicals	2.70	0.05	0.00	0.00	0.00	0.05
	Slurry delivery	0.47	0.00	0.00	0.00	0.00	0.00
	Direct N_2_O from soil	0.00	0.00	0.00	0.00	0.00	0.36
	Indirect (volatisation) N_2_O	0.00	0.00	0.00	0.00	0.00	0.07
	Indirect (leaching) N_2_O	0.00	0.00	0.00	0.00	0.00	0.08
Total first year cutback and fertilisation		3.92	0.10	0.00	0.00	0.00	0.61
Harvesting, maintenance and fertilisation	Forage harvesting	34.14	2.21	0.00	0.00	0.00	2.23
	N_2_O emissions from leaf fall	0.00	0.00	0.00	0.01	0.00	2.73
	Slurry spreading (diesel)	3.21	0.21	0.00	0.00	0.00	0.21
	Slurry delivery	18.69	0.05	0.00	0.00	0.00	0.05
	Direct N_2_O from soil	0.00	0.00	0.00	0.01	0.00	2.85
	Indirect (volatisation) N_2_O	0.00	0.00	0.00	0.00	0.00	0.57
	Indirect (leaching) N_2_O	0.00	0.00	0.00	0.00	0.00	0.64
Total harvesting, maintenance and fertilisation		56.04	2.46	0.00	0.02	0.00	9.27
Storage and fugitive emissions	Storage (unloading)	21.94	1.42	0.00	0.00	0.00	1.43
	Storage (re-loading)	46.23	2.99	0.00	0.00	0.00	3.01
	Storage (methane)	0.00	0.00	0.00	0.00	0.00	0.00
Total storage and fugitive emissions		68.16	4.41	0.00	0.00	0.00	4.44
Total transport diesel consumption		55.94	3.62	0.00	0.00	0.00	3.65
Total non-CO_2_ combustion emissions		0.00	0.00	0.02	0.02	0.00	6.42
Termination of crop	Diesel fuel	18.07	1.17	0.00	0.00	0.00	1.18
	Agrochemicals	0.78	0.01	0.00	0.00	0.00	0.02
Total termination		18.85	1.18	0.00	0.00	0.00	1.19
Total carbon sequestered		0.00	−37.21	0.00	0.00	0.00	−37.21
Grand total (including carbon sequestration)		207.25	−25.19	0.03	0.04	0.20	−11.05
Grand total (excluding carbon sequestration)		207.25	12.02	0.03	0.04	0.20	26.16

#### Storage and Transport

For outside storage, 0.11 and 3.3 l diesel/t are required for unloading and re-loading the chips after 6 months [78]. The wood chip is expected to dry from 50 to 30 % MC during the storage phase. Transportation of the wood chips to the end user (100 km round trip, with one trip empty) then has a fuel requirement of 68 l/ha each harvest or 0.02 l/t km travelled. The calculations are based on a 44 GVW truck, with a 58 % load rate of a total payload capacity of 28.5 t (assumed bulk density of wood chips at 50 % moisture content of 240 kg/m^3^ and a truck volume of 69 m^3^ [79]).

#### Energy Conversion

The Milne equation and Phyllis database (untreated wood, willow) was used to predict the lower heating value of the wood chips [80]. The LHV is the energy released on combustion of a given quantity of fuel excluding the heat obtained by condensing the water vapour produced by its combustion [81]. Changes in the LHV caused by changes in moisture and ash contents of the wood chips are also included.

The wood is combusted in a specialised wood chip boiler with an efficiency of 90 %, similar to those used for domestic use or small–medium scale in district heating schemes. For example, Strebel Taurus wood chip boilers [82] have an efficiency of up to 95 % in smaller models (13–98 kW) and >90 % efficiency in larger scale boilers (42–360 kW). It is assumed that 1.1 % of the total output energy is required for start-up [42]. Ash disposal is assumed to incur negligible environmental impacts. Carbon dioxide emissions from biomass combustion are considered ‘carbon neutral’, though emissions of methane and nitrous oxide are both 0.005 kg per GJ of wood combusted, respectively [83].

Emission factors for diesel fuel and other fossil fuels are derived from current GHG reporting emission factors [84]. The level of GHG mitigation, or greenhouse gas savings, are based on a modern natural gas boiler, also with an energy efficiency of 90 % [85].

#### Termination

After 23 years, the model estimates that a total yield of 360 t/ha is removed from the site, although crop termination can occur before this if new improved varieties become available or if the yields decline due to age or other factors. The method of termination of the crop depends on the age and size of coppice stools and the intended subsequent land use, but the overall goal is to remove or decompose the root structure to prevent further growth and permit future soil cultivation [86]. Assuming that the old SRC crop will be replaced by another, the fastest method of removing old stools is to grub out stumps using a narrow bucket that is attached to a digger [86]. Stump harvesting and extraction, which involves lifting and tearing of tree stumps and roots, is expected to consume diesel at 12 l/h, requiring 15 h/ha or 180 l/ha [54]. The industry expert advised that now it is more common to use a stump harvester, or forest mulcher, of which usually two passes are required to sufficiently destroy the stools. This method has the advantage of speed of work, and the land is available for immediate replanting [86]. It was not possible to collect primary data for mulching process nor could it be provided by the industry expert; therefore, it was estimated from literature. Mulchers are usually very large machines, with a power rating of between 500 and 750 kW and an estimated work rate of 1.5 ha/h [87]. Assuming a high power take-off (80 %) for the highly intensive work, a fuel consumption rate of 113–157 l/ha is required for one pass.

#### Carbon Sequestration

Carbon sequestration under SRC depends on the direct land use change that has occurred. A more detailed uncertainty analysis of the effect of carbon sequestration on the GHG savings of willow is provided in [70]. In this study, carbon sequestration is based on conversion of arable land to willow and uses data from Hillier et al. [81], who deduced the following characterisation of sequestration under SRC during this change in land use:

$$ {C}_{input}=8.01\left(0.5+0.5\left(1-{e}^{-0.23 Yield}\right)\right) $$

where *C*_input_ is the total carbon sequestered (t C/ha) over the lifetime of the crop with a specified average yield. The equation predicts a total C sequestration of 8 t (29 t CO_2_/ha) over the lifetime of the crop. The model is less reliable for crops under 5 years old and does not include the addition of organic fertiliser to the soil during cultivation. It is not yet well understood how much carbon is retained when the long-term energy crops are terminated, and this is rarely discussed in LCA studies on energy crops [88]. Currently, there are no studies examining this in SRC, even though termination of the crop will likely lead to the decomposition of roots and stumps, releasing the accumulated carbon as CO_2_. It is possible that if the site is then re-planted with willow, then the previous level of sequestration could be restored; however, it will reach a similar saturation point [55]; therefore, the sequestration will only be accounted for in the first rotation.

### Sensitivity Analysis

Finally, a sensitivity analysis will provide some indication of the influence of the most important assumptions in the LCA [89]. The effects of dry matter losses and methane emissions from storage are explored by changing the value of the parameters whilst others are held constant [90]. Dry matter losses will range between 1 and 27 % as is the range reported in the literature (Table 1). Emissions of methane from the wood chip stack will be tested between 0 and 9 % of the carbon within the wood chips being anaerobically degraded to methane and ultimately leaving the stack. Emissions of CO_2_ from decomposition in the stack and combustion are considered to be carbon neutral.

## Results and Discussion

### GHG Emission Savings from SRC: Base Case

The LCA study calculates a total gross GHG emission of 27.3 kg CO_2_ eq./MWh generated from SRC willow chips or 6.8 g CO_2_ eq./MJ in biomass, excluding carbon sequestration, assuming zero dry matter losses, and excluding any potential GHG emissions from storage. Comparing this to a GHG emission of 516 kg CO_2_ eq./MWh from natural gas-generated heat boiler (90 % efficiency), it equates to a GHG emission saving of 95 %. The energy ratio of the system is 0.05 MJ/MJ, or 19.31MJ_out_/MJ_in_, which is much lower (95 % saving) than most conventional fossil fuels (e.g. 1.11 MJ/MJ for natural gas [91]). These results refer to a lifetime of 23 years: If for some reason the crop is terminated earlier, for example after 10 years, the gross GHG emissions are almost doubled: 43.1 kg CO_2_ eq./MWh. A sensitivity analysis shows that a loss of yield of 1, 10 and 20 % increases the GHG emission savings by 0.4, 5 and 11 %, respectively. Adjusting the model to instead compare a dedicated electrical generation, with 20–40 % conversion efficiency in the biomass plant [92] and 40 % in the natural gas plant [93], gives a GHG saving of 89 to 95 %, without carbon sequestration. Overall, the results of this study suggest that the use of SRC for heat or power causes the release of far fewer GHG emissions per MWh than natural gas.

Another study compared US grid electricity with SRC willow gasification and reported GHG savings of 95–96 %, and 10 % co-firing with coal gave 9.9 % savings [94], including carbon sequestration (14 t CO_2_ eq./ha). When carbon sequestration is included, a net negative GHG emission of −10.0 kg CO_2_ eq./MWh or 102 % saving is achieved. The result is based on the assumption that 29 t CO_2_ eq./ha are sequestered under the crop over the 23-year lifetime, attributing each tonne harvested over the lifetime with −75 kg CO_2_ eq. The build-up of organic carbon under the crop changes a net GHG emitting to a net-negative bioenergy system, though this is dependent on how much carbon is effectively locked away. According to the LCA model developed, the turning point is 5.6 t C/ha or 20.5 kg CO_2_ eq./ha for the heat system to be GHG neutral. If the sequestration late is lower, then SRC still achieves very large GHG savings compared to natural gas.

The results highlight the importance of understanding the full extent at which carbon is sequestered under SRC willow and to discern how it changes after the termination of the crop. Data is lacking for this in energy crops. One unpublished study by Duffosé et al. [95] terminated a 20-year-old stand of *Miscanthus* in France that had achieved a sequestration rate of 45.1 t CO_2_/eq./ha under the crop or an increase of 9.6 % compared their annual land control. After ploughing, samples from gas chambers measured an accumulated loss of 6 t CO_2_/ha from the soil over the preceding 10 months, even if the land was re-cultivated, suggesting that there is some loss of organic carbon due to disturbance and destruction of the biomass. After termination, it is possible that some finer root components could remain at depth; however, the rate at which these carbon pools are oxidised level depends on the level of tillage the site receives in the following years. If the site is replanted, the previous level of sequestration could be restored [55], but if instead an arable system follows, then it is hypothesised that a great loss of carbon will occur [81]. It is recommended that further exploration is required on this stage of the life cycle of energy crops to fully understand the GHG mitigation potential of SRC.

### Breakdown of GHG Emissions: Base Case

**Fig. 2:**
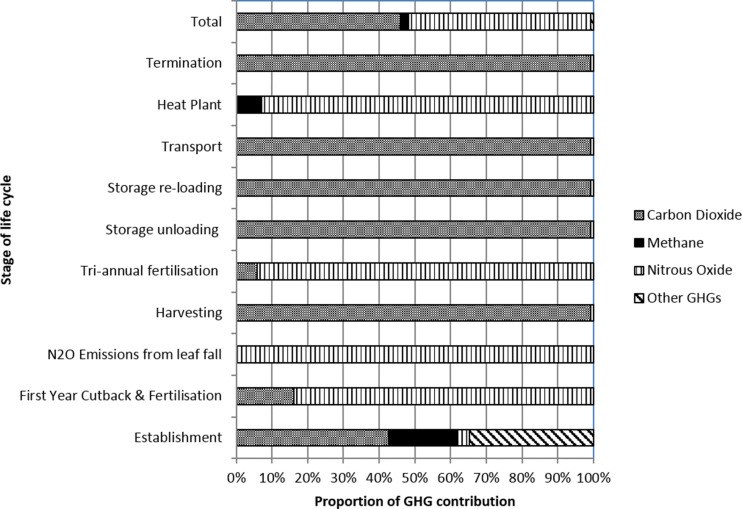
Breakdown of GHG emission sources in each stage of the SRC life cycle

**Fig. 3:**
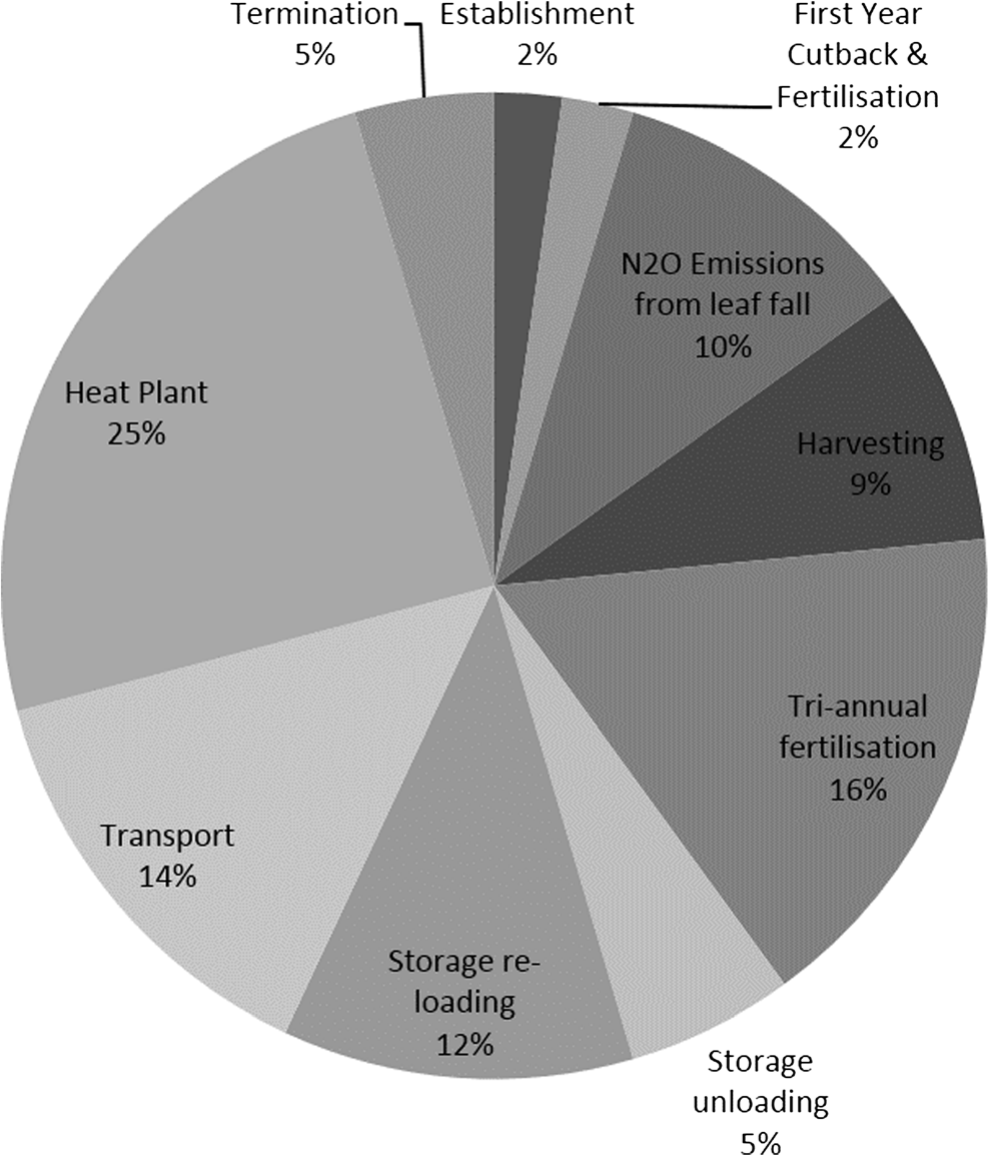
Breakdown of the total GHG emissions from each stage of the SRC life cycle

Figure 2 shows the breakdown of GHG emissions for each stage of the supply chain, and Fig. 3 shows the contribution of each GHG gas to the total result. Table 3 provides a thorough breakdown of the primary energy requirement and GHG emissions from each contributing component. ‘Site establishment’ represents 2.2 % of the GHG emissions per MWh; therefore, despite establishment being relatively expensive and energy-intensive phase, it has a negligible contribution to the life cycle of the crop after being shared between the entire yields of the site. If the crop was terminated after 10 years, site establishment would represent 2 % of the emissions per MWh. These results show that an intensive establishment phase does not compromise the GHG savings from the system, and it is vital to ensure successful yield of the crop over the following years.

Delivery of cuttings contributes 0.5 % of the footprint per MWh or a negligible contribution. Herbicide application contributed 0.3 % of the total emissions per MWh, and it may be likely that reduced applications could *increase* the GHG emissions per tonne of biomass grown due to increased weed competition having a negative impact on yield. This is also suggested by Berry et al. [96] regarding the use of fungicides in wheat.

Figure 2 shows that N_2_O emissions dominate (51 %) the GHG emissions from entire life cycle GHG emissions. These emissions are prevalent in the biomass combustion phase, site fertilisation with slurry and from leaf fall, these stages contributing to 25, 16 and 10 % of the total GHG emissions per functional unit, respectively (Fig. 3). These are discussed in the following subsections.

#### Biomass Combustion

Biomass combustion leads to the release of a number of gaseous and particulate emissions, namely CO_2_, CO, CH_4_, H_2_, unburnt hydrocarbons, particulate emissions and soot particles [97]. Here, only the CO_2_ component of the flue gas is considered to be ‘neutral’; non-CO_2_ emissions (CH_4_ and N_2_O) also occur during biomass combustion. Emissions of CH_4_ result from incomplete combustion [98] and N_2_O from the direct conversion of nitrogen in biomass boilers [99]. Emissions of both are dependent on the temperature of combustion and are highly uncertain [98]. Here, emissions assume an emission factor or 0.005 kg/GJ biomass for both gases [83]. Alternative estimates include 0.03 kg CH_4_/GJ and 0.004 kg N_2_O/GJ from the IPCC [98], 15 kg CH_4_/t biomass [100] and a conversion of 0.4 % of N in spruce wood to N_2_O and 7.2 % for beech wood [99]. In summary, these emissions are highly variable and not very well documented.

It is interesting that in the RED methodology, for the calculation of GHG emission savings from biofuels sets a default value of ‘zero’ for emissions from combustion (*eu*, point 12, Part C, Annex V [3]). Here, these emissions contributed 25 % of the final emissions per MWh, suggesting that the assumption in the RED is incorrect. This was also observed by Hagberg et al. [83], who examined the applicability of the RED to wood pellet supply chains. Likewise, the Solid and Gaseous Biomass Carbon Calculator, which was developed to facilitate sustainability reporting for Renewable Obligation-accredited generators [101], also excludes non-CO_2_ emissions from combustion [102]. Despite there being a high variability in emissions between biomass feedstocks and conversion equipment options, excluding them from the systems boundary is a substantial omission from the life cycle of the bioenergy crop.

#### Site Fertilisation with Slurry

The delivery of slurry and direct and indirect N_2_O emissions from soil contributes 5 and 17 % of the total footprint, respectively. It is possible that detailed modelling of N_2_O emissions could refine the result. The final GHG emissions would be or 0.03 % more if the manure was transported 40 km instead of 10 km to deliver to site, though it would become more expensive. The GHG emissions for slurry delivery (10 km) were estimated at 2 kg CO_2_ eq./m^3^ or 0.4 kg CO_2_ eq./kg N. This is comparable to ammonium sulphate (AS, 0.59 kg CO_2_ eq./kg N), so the GHG emissions would not be severely compromised by using AS instead, although it may be more financially viable to use organic fertilisers. Artificial fertilisers will also require delivery to the farm; however, that is expected to be far more efficiently delivered.

The potential for SRC willow as a phytoremediation method for organic substances containing trace heavy metals have led to the promotion of SRC as a multifunctional crop [103]. It is anticipated that the accumulation of substances in the willow crop can be removed in the ash through flue gas cleaning [55]; however, this should be explored further to avoid issues during combustion. Application of manures can also act as a soil amendment and contribute to carbon sequestration [104], which has already been identified as a key contributor to the GHG emission balance of the crop.

Disadvantageous environmental impacts from organic fertilisers include eutrophication of water systems from run-off, erosion and leaching or from volatisation of ammonia from storage and application [105]. In the UK, nitrogen vulnerable zones (NVZs) highlight sites that must comply with legal applications of nitrogen-based fertilisers to avoid environmental damage [106]. In some instances, leakage of phosphorus or potassium can trigger eutrophication where water systems are saturated with nitrogen [105]. It is important to monitor the P and K status of the soil over long periods of manure addition, although applications of 60–120 kg N/ha should be within acceptable application rates [103, 107]. Volatisation of ammonia is also implicated with acidification of soils and human health problems [105].

#### Emissions from Leaf Fall

**Fig. 4:**
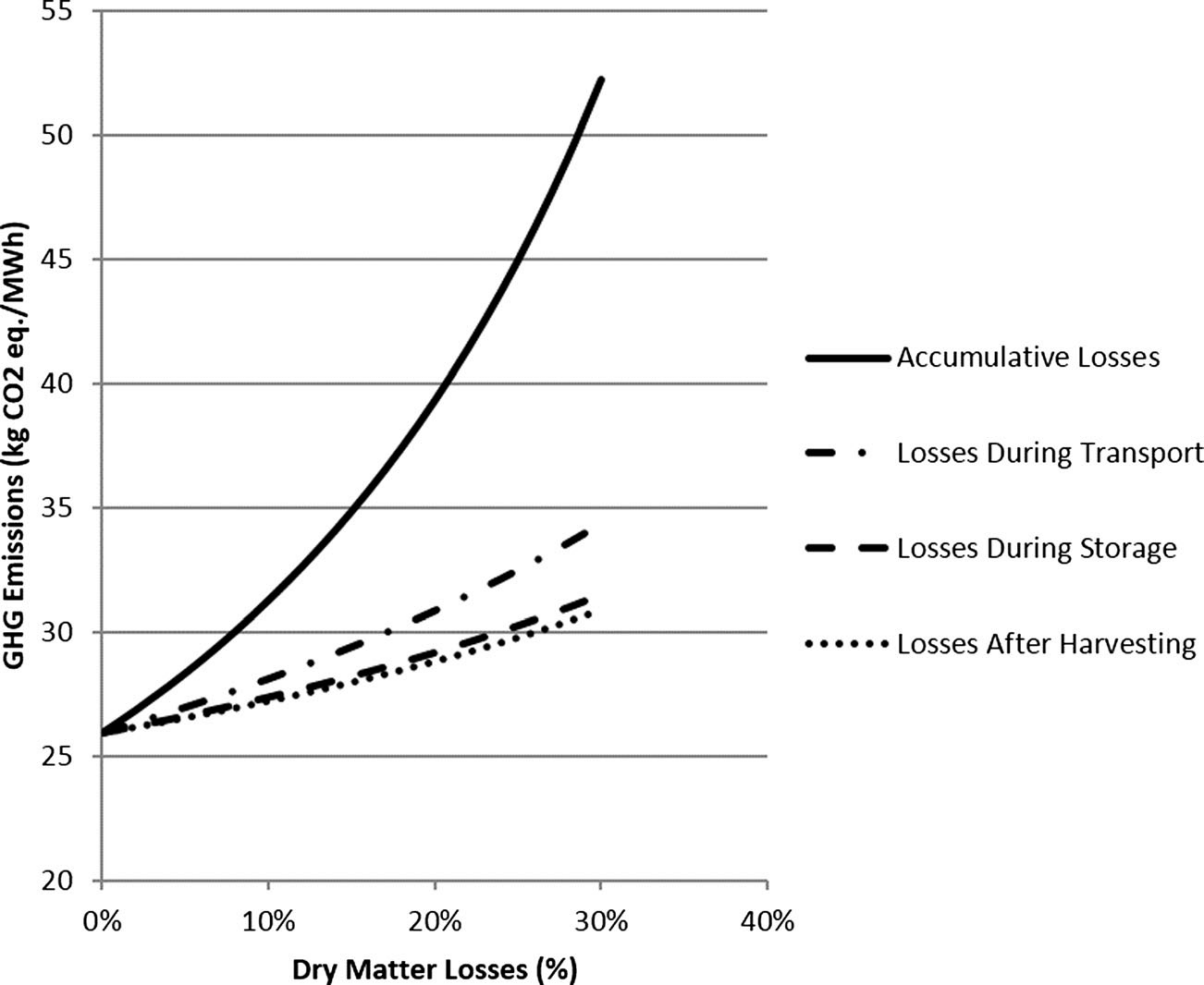
Impact of dry matter losses on the GHG emissions of SRC-generated heat

Nitrous oxide emissions from leaf fall are another main single contributor to the life cycle of the crop (Fig. 4). Here, a total release of 2.2 t CO_2_ eq./ha over the 23 years of the plot is calculated. Heller et al. [47] is one of the few LCA studies to also include this input, assuming an emission of 7.3 t CO_2_ eq./ha, based on a deposition of 3.2 t/ha of leaf material per year. There is currently research taking place in the UK, such as the CarboBioCrop Project (http://www.carbo-biocrop.ac.uk) and the Ecosystem Land Use Modelling and Soil C Flux Trial (ELUM, http://www.ceh.ac.uk/sci_programmes/elum-project.html), now continuing as Measurement and Analysis of bioenergy greenhouse gases: Integrating GHGs into LCAs and the UK Biomass Value Chain Modelling Environment (MAGLUE). The projects aim to measure changes in GHG emissions from soil under energy crops, including recording of leaf matter inputs to soil and soil carbon fluxes due to land use change. The results of current research will help to refine the figures used in here.

### Sensitivity Analysis: Impact of Dry Matter Losses During Storage

Fuel requirements for the storage phase contributed 17 % of the total GHG emissions per MWh, including unloading, stack building and reloading chips for transport. During storage, it is assumed that the biomass dries from 50 to 30 %, according to the Milne equation [80], equating to an increase from 7.8 to 11.3 GJ/t. In theory, the storage phase has net gain of 3.4 GJ/t, including handling stages. This is currently based on theory rather than experimental data and does not include losses from the storage phase. Figure 4 shows the impact of dry matter losses that occur during the supply chain. Although losses mean that less material must be handled and processed, the total GHG emissions per MWh increase because the net yield of energy per hectare decreases. The LCA model estimates that a 1, 10 and 20 % loss of dry matter during storage causes a 1, 6 and 11 % increase in GHG emissions per MWh. The losses reduce the net energy yield from land: A dry matter loss of 10 % from the storage phase reduces the net energy yield from 137 to 124 GJ/ha/year. Figure 4 also shows that as the GHG emissions from invested processing and handling accumulate, greater penalties arise if biomass is lost at the later stages of the supply chain.

**Fig. 5:**
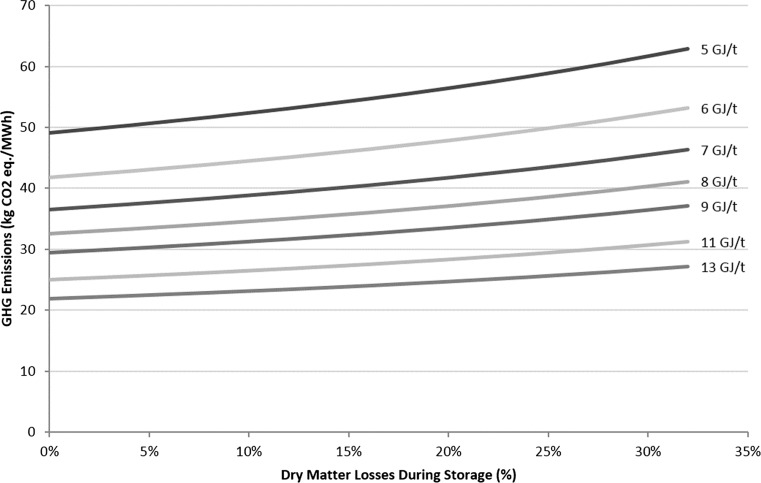
Impact of dry matter losses and final LHV of the SRC wood chips after storage on the GHG emissions from SRC-generated heat

The GHG emission results (per MWh) are also affected by the extent of biomass drying during storage, with less efficient drying resulting in higher GHG emissions per MWh (Fig. 5). A number of studies have shown that the net energy change in the biomass due to storage is most often negative [108]. The model estimates that just a 4 % storage loss of dry matter is enough to have a net loss of energy from the storage phase, even if the chips dry efficiently to 30 %. Alternatively, if the wood chips do not dry or get wetter due to poor weather or storage conditions, then a loss of energy will occur, with or without dry matter losses.

Factors affecting the LHV may not be as straightforward as moisture effects alone, as it also affected by ash or volatiles changes during storage. Ash compositions tend to increase due to the decomposition of biodegradable components of the biomass, resulting in a decrease in combustible proportion of the biomass [109]. Other studies showed a 5–6 % increase in ash content of poplar crown, and stem chips was observed after 6 months of outside storage [24] and a 7 % increase in ash content in 6 m high piles of willow wood chip [26], though others report minor changes that could be described by natural variation [10, 80, 110]. Such small changes in ash content would make a negligible change to the LHV, however. Despite the reduction of moisture content, a loss of volatiles from the biomass results in a loss of embodied energy [10]. Graham et al. [111] observed a loss of 66 % of the volatiles after air-tight storage of forest residue chips in a laboratory, however does not translate the results into a change in LHV; therefore, some more experimental data may be needed to examine this effect.

Dry matter losses during storage may be reduced by storing wood in uncomminuted form [15, 112, 113]. Uncomminuted biomass has the benefit of more efficient forwarding, transportation and can benefit from large-scale chipping [114], although the handling costs of uncomminuted wood is generally higher than for wood chips [24]. Even if wood was stored in whole chunks, it would be necessary to store a buffer supply of chips [16]. Alternatively, if wood chip storage cannot be avoided, it is believed that the transfer of heat and moisture from the wood chip stack is dependent on the rate at which the moisture can diffuse through the stack [16]: Smaller piles of chips or those made from larger particles, such as billets, should avoid rapid heat generation seen in larger stacks [9, 13, 16, 115]. Some studies examine covering biomass piles to protect them against rain; however, it is generally found that an adequate airflow is necessary for effective drying and impervious covers could cause mould formation and composting [10, 116]. Manzone et al. [117] found that losses were minimised and moisture content improvements were achieved with a fleece cover; however, the authors suggest that uncovered storage is a cost-effective option as the costs of covering are greater than any benefits of reduced dry matter losses.

### Sensitivity Analysis: Impact of Methane Emissions from Storage

As in composting, the process of degradation begins with the readily available storage of nutrients in the form of starch and fat that are released after the comminution process [109]. Here, the authors note that the losses of carbon in the form of methane are also accounted for as dry matter losses. The process is associated with a heat increase and the rapid depletion of oxygen, meaning that anaerobic conditions prevail in the core parts of the stack, leading to the release of methane [40]. The LCA model shows that the GHG emission results are extremely sensitive to emissions of methane from the wood chip stack: A release of 1, 2 and 3 % of the carbon within the biomass in the form of methane increases the GHG emissions by 206, 413 and 618 %, respectively. This equates to a reduction in GHG emission savings (including carbon sequestration) from 102 to 92 %, 81 and 71 %, respectively, with each 1 % increase in carbon conversion to methane increasing the GHG emission per MWh by 55.22 kg CO_2_ eq./MWh. A 1 % conversion rate is equivalent to 2.14 kg CH_4_/t stored wood. In summary, the GHG emissions from SRC wood chip supply chains are highly sensitive to methane emissions from the storage phase, though with small emissions, the SRC system still achieves GHG emission savings compared to natural gas. The model calculates that GHG savings of up to 60 % can still be achieved if up to 4 % of the carbon is lost through anaerobic degradation. After 9 % of the carbon is lost via this route, the GHG emission savings are virtually zero compared to natural gas boiler.

It is questionable whether landfill models for biodegradable material are relevant to wood, as there is evidence that it slowly decays in landfill due to the recalcitrant nature of the material [118, 119]. Wood has a ‘half-life’ of 20–40 years in landfill, and one hypothesised methane yield is 0.013–0.022 g methane/g dry wood [32], equating to 1.1 kg/t wood (50 % MC). If this is entered into the model, then the GHG emissions are estimated at 34.9 and −2 kg CO_2_ eq./MWh, including and excluding carbon sequestration, respectively, or a 93 or 100 % GHG saving; however, the methane emissions represent 25 % of the footprint. Wihersaari et al. [12] hypothesised that methane emissions would occur from wood chip stacks during the first 2 months at a rate of 24 g CH_4_/m^3^/day or a conversion of approximately 1.5 % of the carbon to methane, equating to an emission of 116.9 or 80 kg CO_2_ eq./MWh including and excluding carbon sequestration, respectively, or a 77 or 84 % GHG saving.

## Conclusions and Outlook

A life cycle assessment was performed on the cultivation and management of short rotation coppice willow that was harvested, stored and combusted in a small-scale heating boiler. The study was designed to reflect current commercial cultivation of SRC, including the propagation of cuttings and termination of the crop at the end of life. The results of the study show that SRC generally provides very high GHG emission savings of over 90 %, compared to natural gas, and sequestration of at least 7 t CO_2_ eq./ha under the crop means that it is net zero GHG emission system. There are some uncertainties in the LCA results, regarding non-CO_2_ emissions from combustion, N_2_O emissions from leaf fall and the extent of carbon sequestered under the crop. These all contribute a large proportion of the life cycle GHG emissions from cultivation of the crop. It is recommended that further research is performed in order to refine the numbers used in this study.

Dry matter losses increase the GHG emissions per MWh, as well as reducing the net energy yield from land. This study shows that a loss of 4 % of the dry matter or a wetting of the wood chips can lead to a net energy loss from the storage phase. The LCA model that calculated a 1, 10 and 20 % loss of dry matter during storage causes a 1, 6 and 11 % increase in GHG emissions per MWh. It is recommended that alternative storage options are explored to reduce the impact of losses from the supply chain stage.

It is possible that the biological processes responsible for dry matter losses also lead to anaerobic decomposition within the stack, which can lead to the production and emission of methane. The LCA model showed that if 1 % of the carbon within the stack undergoes anaerobic decomposition, then the GHG emissions per MWh are tripled. Therefore, methane emissions from the storage phase are highly uncertain yet have the potential severely compromise GHG savings from woody supply chains. It is recommended that further research is performed to examine the evolution of methane within wood chip stacks and to test whether this can be avoided by alternative methods of storage (small vs. large stacks) or storage in uncomminuted forms.
